# Quantitative Microbial Risk Assessment and Opportunist Waterborne Infections–Are There Too Many Gaps to Fill?

**DOI:** 10.3390/ijerph15061150

**Published:** 2018-06-01

**Authors:** Richard Bentham, Harriet Whiley

**Affiliations:** College of Science and Engineering, Flinders University, GPO Box 2100, Adelaide 5001, Australia; Harriet.Whiley@flinders.edu.au

**Keywords:** opportunistic pathogens, QMRA, risk assessment, public health, manufactured water systems, drinking water, *Mycobacterium avium*, *Pseudomonas*, non-tuberculous mycobacteria

## Abstract

Quantitative microbial risk assessment (QMRA) is a relatively new approach in identifying health risks associated with the ubiquitous presence of pathogens and opportunists in the human environment. The methodology builds on experimental and meta-analytical data to identify measurable factors that contribute to, and can quantify, the likely extent of disease given a particular exposure. Early modelling was particularly focused on food-borne disease, and subsequently water-borne disease, with the emphasis focused on ingestion and its role in enteric disease. More recently, there has been a focus on translating these principles to opportunist waterborne infections (OWI) with primary focus on *Legionella* spp. Whereas dose and susceptibility are well documented via the ingestion route of exposure there is considerably less certainty regarding both factors when understanding *Legionella* spp. and other OWI. Many OWI can arise through numerous routes of transmission with greatly differing disease presentations. Routes of *Legionella* spp. infection do not include ingestion, but rather aspiration and inhalation of contaminated water are the routes of exposure. The susceptible population for OWI is a vulnerable sub-set of the population unlike those associated with enteric disease pathogens. These variabilities in dose, exposure and susceptibility call in to question whether QMRA can be a useful tool in managing risks associated with OWI. Consideration of *Legionella* spp. as a well-documented subject of research calls into question whether QMRA of OWI is likely to be a useful tool in developing risk management strategies.

## 1. Introduction

In the early 1990’s Quantitative Microbial Risk Assessment (QMRA) was developed as a tool to understand the likely outcomes of microbial exposures in food [1,2]. This innovative approach has been widely accepted as a major development in protecting public health. Subsequently, QMRA became a useful tool in determining threshold limits for microbial contamination in manufactured food products. More recently the QMRA principles were applied to potable water supplies [3,4]. QMRA were developed for a range of enteric bacterial and protozoan parasites in water distribution systems. It is worthy of note that all of these useful developments were focused on the ingestion or oral/fecal route as the primary mode of transmission [2,4]. It has been proposed that QRMA should be developed for other opportunist waterborne infections (OWI) such as *Mycobacterium avium* and *Pseudomonas* species in engineered (potable) water systems [5]. The stated overall objective of QMRA is to inform health risk assessment and management practices.

QMRA relies on both laboratory and field data to identify an infection threshold. Infectious dose is one significant element in the calculation. The number of organisms required to induce infection has been calculated from historical data and in many applications, is quite robust. In developing QMRA the response of an exposed population has mostly (but not exclusively) been compiled from field data. From these compiled data the likely morbidity and mortality of a given population can be predicted given the exposure scenario, exposed demographic and ingestion. In terms of disease burden this is commonly summarized as a Disability Adjusted Life Year (DALY). Factors used to make these predictions include source of contamination, consumption, dose-response and frequency of exposure. These factors can then be used to quantify the different stages of a risk assessment and a management strategy [4,6].

*Legionella* spp. is a well characterized and researched organism that can serve as a representative OWI. Clinical presentations of disease and routes of exposure for *Legionella* spp. infection are less diverse than other OWI, such as *Mycobacterium avium* complex (MAC) [7]. This allows *Legionella* spp. to be used as a simple model for consideration of QMRA as a useful risk management tool for other OWI. The authors are not aware of published QMRA for other OWI in potable water systems.

**Figure 1:**
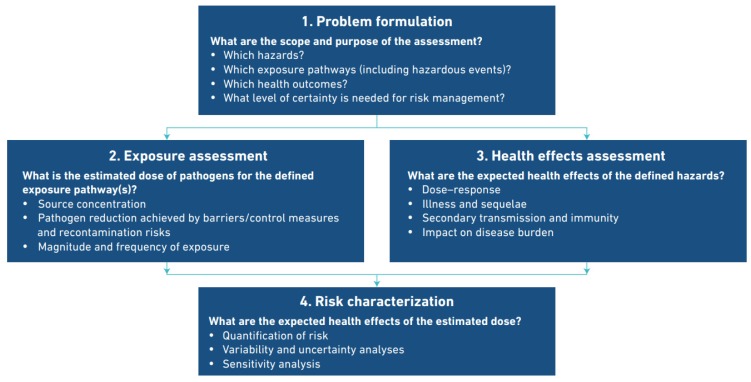
World Health Organization accepted framework for water related QMRA [4].

Recently there have been attempts at developing a QMRA framework to quantify risks from *Legionella* spp. contamination in potable water systems [8,9,10,11]. Research gaps in developing such a framework have been identified [12,13]. These gaps include source of contamination, exposure routes, dose-response and exposure assessment, variable host susceptibilities and uncertainties. Given the uncertainty in all the factors used to develop a QMRA framework it is questionable whether a meaningful model can be arrived at for contraction of *Legionella* spp. infections. The accepted framework for water related QMRA is reproduced from the World Health Organization document [4] and shown in Figure 1: This paper discusses the uncertainties surrounding these factors and their potential for use in a meaningful QMRA for *Legionella* spp. infections and their implications for other OWI.

## 2. Clinical Presentations

The potential for *Legionella* spp. exposure to engender two different clinical presentations is also a reality to consider. Armstrong and Haas [8] developed a QMRA model for *Legionella* spp. infection during spa pool exposure. The study neglected to consider whether the outcome of exposure was Legionnaires’ disease or, the milder syndrome, Pontiac Fever. Documented reports show that one or other, and very occasionally both disease presentations may be associated with a single spa pool or cooling tower [14,15]. This calls into question the thresholds identified by Armstrong and Haas [8] to determine clinical and sub-clinical infection. This is especially true as doubt exists whether Pontiac fever is an infection or not, and some evidence demonstrates that the syndrome is not limited purely to *Legionella* spp. exposures [16,17].

## 3. Sources of Contamination

A wide range of different sources of *Legionella* spp. contamination have been identified. Cooling towers, spa pools, and potable water systems are the most commonly identified sources [18]. However, a recent systematic literature review conducted by van Heijnsbergen et al. [19] identified thirty other potential sources including natural waters, medical respiratory equipment, construction activities, ice machines and hoses. The European Working Group for *Legionella* spp. infections (EWGLI) (2017) acknowledges that potable water and cooling towers are likely to contain low levels of *Legionella.* It is recommended that a positive *Legionella* spp. detection less than 1000 CFU/L requires no response other than to ensure biocide levels, temperatures etc are within targets. Whereas, positive detections above 1000 CFU/L in 80–90% of samples warrants a risk assessment to determine if remedial actions (including disinfection) are required. The ubiquitous nature of *Legionella* spp. in the environment raises the question as to why the number of Legionnaires disease cases are comparatively low. “*…one fundamental paradox of legionellosis epidemiology is that if these microbes are environmentally abundant so that exposure must be almost ubiquitous, why is disease uncommon? What are we missing?*“ [20].

The diversity of sources also illustrates that the nature of their contamination is similarly diverse. Water supply quality is often more controlled that water quality within buildings. Factors that play an important role in *Legionella* spp. proliferation in manufactured water systems include temperature, nutrients, flow rate and disinfectant residual [5]. These also influence biofilm formation which is acknowledged as the principle multiplication point for *Legionella* spp. and other opportunist pathogens. However, the nature of biofilm formation, extent, and location within the system is very much determined by the source construction [18].

Variations in the water supply and quality are common both spatially and temporally. As water quality has a known influence on microbial colonization, including Legionella, supply quality is a critical parameter that is unpredictable even within a single source. Changes to supply water quality have been identified as the single major contribution to elevated numbers of Legionnaires’ disease cases in Flint (MI, USA) in 2016 [21].

Changes in the environmental conditions within a water supply can also result in a distinct shift in *Legionella* spp. populations. This was highlighted by a study of hot and cold water samples collected from a single potable water distribution system in Braunschweig (Germany). This study demonstrated that the composition of *Legionella* spp. was distinctly different in the hot and cold water and this difference was observed throughout a seasonal cycle [22]. It is worth noting that some sources are associated with specific *Legionella* spp. and not others. For instance, outbreaks of disease associated with cooling towers are almost exclusively confined to *Legionella pneumophila* serogroup 1 (SG1). Other species of *Legionella* spp. tend to be associated with infections from devices and equipment associated with high risk populations [18].

This diverse range of sources combined with a relatively long incubation period (1–21 days) has led to considerable uncertainties in identifying either the source or the organism responsible in outbreak scenarios [18]. For example, *Legionella* spp. may be detected in a suspected source during an outbreak investigation but genotypical analysis may find it to be different to cause of the outbreak [23]. Identifying outbreak sources is also complicated by the limitations of the culture method of detection which does not detect viable but non-culturable *Legionella,* resulting in false negative test results [24].

These combined factors mean that a single quantifiable or estimable source of contamination by *Legionella* spp. is source specific and in some instances likely to be unique. This suggests that risk assessments should be equally site-and source-specific to be effective.

## 4. Exposure Route

There are two well documented exposure routes, inhalation of contamination aerosol and aspiration of contaminated sources. Assessing aerosols as a potential inhalation route is complicated by the lack of correlation between *Legionella* spp. concentration in contamination water and *Legionella* spp. concentration in the air as a consequence of aerosol production [25]. This in part may be explained by the differences in aerosol size generated by different water uses and the effect this has on *Legionella* spp. transmission to the lower respiratory system [26]. In addition there may be substantial differences in exposure from an aerosolized water droplet compared to a vesicles containing *Legionella* spp. expelled from a protozoa. Depending on the host these vesicles can vary in size and the concentration of *Legionella* spp. they contain [27,28].

In the instance of aspiration the volume of contaminated water entering the lung is determined by the source and the susceptible person exposed [4,18,29]. It is clear that potable water is the principle, if only, source of aspiration of contaminated water. Such devices as cooling towers and other remote aerosol producing devices can therefore be ruled out as putative sources. More confounding is that sources such as showers, basins, and spa pools are credible sources of aspiration of contaminated water. At the same time they are equally credible sources of aerosol inhalation [18,29].

Clearly aspiration must be considered as the only route of exposure from some sources, such as ice machines. Equally aerosol inhalation must be considered as the only route of exposure for other sources. This is particularly true of cooling water systems. Case control studies have demonstrated that cooling towers are a source of Legionnaire’s disease and can cause disease at considerable distance from the source. In these instances, aerosol inhalation is the definitive exposure route [15,30].

## 5. Dose Response

Multiple factors must be considered in determining a dose response for *Legionella*. These include the dose delivered, the virulence of the organism, and the susceptibility of the exposed population. In the case of Legionnaires’ disease all three of these factors are not readily quantifiable.

### 5.1. Dose

Doses delivered by the aspiration route and aerosol inhalation route are not comparable. Aspiration involves relatively large volume droplets of contaminated water entering the lung. Aerosol inhalation requires very small volume droplets to enter the lungs. Arguably, aspirated droplets are likely to have lower concentrations of cells per mL than aerosol in equivalent volumes. Conventional thought is that concentration of cells in aerosol during travel is a contributing factor to disease. Travel of aerosol results in concentration of the droplet contents. As a consequence the same volume of droplets from a single source may contain different numbers of bacteria depending on whether they are aerosolized or not [31]. If this is accepted, then aerosol introduces smaller volumes of more concentrated cells per mL [29].

The disparity between volumes and concentrations and the behavior of aerosols in transit, considering climatic influences, makes comparison with aspiration impossible. The issue of dose becomes even more confused when considering sources in which both disease presentations may occur [15]. In such cases calculating dose from a water body with a known concentration of infectious cells per mL is not possible. 

Recently, Strydom et al. [32] discussed dose response data for *M. tuberculosis* (TB) infections. They concluded that there was ‘little benefit’ in applying QRMA to TB infections. This opinion was based primarily on the uncertainty surrounding the measurable dose response of aerosolized infectious particles.

### 5.2. Virulence

Previous QMRA have mostly focused on ingestion of enteric pathogen; organisms with known infectious doses and broad population responses. QMRA for *Cryptosporidium* exposure has clearly identified the aggregate risks of exposure of a population to oocysts at different concentrations. In this example the organism is an obligate water borne parasite with a known virulence. As such it lends itself to a QMRA based risk assessment approach to managing risk [4].

*Legionella* spp. is an opportunist pathogen with a diverse range of species causing disease. Virulence is widely variable within species and strains. Edelstein and Metlay [33] reported that there are more than 400 different strains of *L. pneumophila* SG1 alone. Of these identified strains a minority can cause disease. 

Some current research has identified that virulence of a single strain varies depending on the stage in the life cycle of the organism. It is clear that a single strain may be infectious or not dependent upon the stage in a complex life cycle that includes 14 different developmental forms [34]. In addition, replication within an amoeba host has been shown to increase *L. pneumophila* virulence [35]. This has also been demonstrated for other opportunistic pathogens such as *M. avium* [36].

### 5.3. Animal Models of Exposure

Many studies used in developing QMRA for enteric pathogens have used human studies as well as field estimates of exposure to establish both virulence and dose response. Clearly, exposure of human subjects to infectious doses of *Legionella* spp. bacteria to establish a dose response is not an ethically sound option. As a result, much of the data used for exposure models has been conducted on animals under laboratory conditions [9,37]. In these investigations healthy but susceptible animal species (guinea pigs) have been used as exposure models to controlled doses.

This introduces two confounding factors. Firstly, Legionnaires’ disease is widely accepted as a disease of unhealthy individuals (immune-compromised). Secondly, it is also widely accepted that humans are not particularly susceptible to infection. Infection is described as opportunistic [29].

Investigations have established that some animals are not susceptible to disease (mice) even at high dose concentrations [38,39]. This data confirms the differences in susceptibility between species. Similarities in cellular responses to *Legionella* spp. infection between mice and humans has also been reported [39].

Interpolating the results of animal models using intrinsically susceptible species into human exposure scenarios is therefore a major source of uncertainty. Using data from less susceptible species (mice) would be more representative of real world dose responses. Regardless, the data would not be able to represent dose response of an immune compromised sub-set of an otherwise resistant (healthy) population.

Furthermore, animal models are based on exposure to aerosol in a carrier that optimizes lung penetration. It is also reported that routes of exposure for these experiments were not consistent. Both aerosol and intranasal routes of infection have been investigated [40,41]. Clearly this is not the case in real world scenarios. Once again, these models, in some case deliberately, neglect aspiration as a mode of infection [8].

### 5.4. Population Susceptibility

Disease incidence has reported ranges between 0.5 and 2+ notified cases per 100,000 across different jurisdictions [42]. These incidences relate to the entire population. Disease incidence and mortality rates are considerably higher in health care settings and other susceptible populations. Predicting the susceptibility of a given population requires consideration of the demography of those exposed, although gender (with males at greater risk) and age are consistent risk factors [18,42].

The literature identifies that *Legionella* spp. infections are for the most part opportunist. This may not be true for Pontiac fever which may be a hypersensitivity in response to exposure rather than direct infection. Whether Pontiac fever is a disease associated solely with *Legionella* spp. remains in question. Symptomatic similarities to exposure to other aerosolized gram negative bacteria are a documented feature of disease [16,43].

In cases of Legionnaire’s disease a broad range of clinical presentations occur, ranging from mild ‘flu like symptoms to serious pneumonia. A determining factor in disease presentation is the immune status of those exposed. Once again, this definition of susceptibility is very broad. Available data to establish a DALY for *Legionella* spp. infection highlights the high mortality rate (Years of Life Lost, YLL) in susceptible groups as the major contribution (92%) to the burden of disease [44]. This means that the high mortality rate of a susceptible sub-set of the population skews the DALY calculation. Inclusion of DALY data into QMRA is therefore statistically questionable.

Smokers are arguably the most susceptible demographic group. To date there is no available data on the extent of smoking habits and the likelihood of infection. This leaves smoking as a ‘sliding scale’ of susceptibility.

Persons over 50 years old are another identified risk factor. This may be linked to the established declining immune capability with age or to compounding age related health factors. There is no current means of measuring the extent of immune competence in the aged.

Persons who are immune-compromised are also a high risk group. This range of susceptible people encompasses a wide spectrum from cardiac patients to oncology and stem cell treatments. Obviously, the level of susceptibility will be unique to the treatment and the recipient and will remain incalculable.

An additional confounding factor is that in many instances the susceptibility of this immune-compromised group to both aerosolization and aspiration is elevated. Swallowing disorders linked to chemotherapies and neck surgery are accepted elevated risks for infection by aspiration [18]. In these scenarios the dose delivered by a given source cannot be separated between aspiration and aerosolization of contaminated water containing the same number of organisms.

Neonates are also an identified high risk group. Documented infections via both aspiration and inhalation routes have been reported [45,46,47].

Underlying lung disease is another risk factor that adds uncertainty. The inspiration and expiration volumes of patients with chronic pulmonary obstruction disease (COPD) are extremely variable. In any case the inspirational volume will be less than unaffected individuals. What this mean in terms of dose inhalation and expiration is unclear. Whether less aerosol is inhaled, or less expired, or residence time of contaminated water increases (all likely in COPD sufferers) will directly influence what can be considered an infectious dose [48].

## 6. Exposure Frequency

Exposure frequency and duration resulting in disease is very much related to the exposure source. Cooling towers and spa pools are established point sources of Legionnaires’ disease. In instances where data is available it seems a single exposure to contaminated aerosol is sufficient to cause disease. Duration of exposure is poorly documented but review of outbreaks from both sources suggests that infection can result from less than an hour of either direct (physical contact with the source) or indirect exposure (proximity to the source) [15,30,49].

In nosocomial settings the duration of exposure can be crudely defined by the length of stay of the affected individual. However, the exposure frequency can be extremely variable ranging from daily shower use to single use of a contaminated appliance. The nature of the appliance will dictate it’s frequency of use and so determine the duration of exposure [50].

The uncertainties and data gaps necessary for a meaningful QMRA for *Legionella* spp. are tabulated below. The table is based on the World Health Organization (WHO) schematic presented at Figure 1 above. The table highlights the persistent knowledge gaps that are unlikely to be resolved.

## 7. Summary

QMRA has been a useful tool in assisting hazard analysis and critical control point (HACCP)-based risk management programs for food processing and water utilities. It has assisted in providing threshold-based acceptable limits for microbial contamination and source quality for these systems. The considerable uncertainties associated with *Legionella* spp. infections (summarized in Table 1) make it very difficult to capture quantitatively the nuances of human health risk associated with a diverse population of sources, contaminants, exposure routes and susceptible populations. 

An argument for the use of QMRA is that it can be used to identify knowledge gaps and uncertainties that can direct future investigations [6]. In the case of *Legionella* spp. infection the knowledge gaps regarding contamination, consumption, dose-response and frequency of exposure are likely to remain persistent and indeterminable. This is, in part, due to the opportunist nature of *Legionella* spp. infections that dictate susceptibility and exposure as the major influences on health outcomes. Combined with variable virulence and multiple sources, *Legionella* spp. infections pose a different and more complex scenario than the relatively simple case of infection via ingestion of pathogens of known virulence and measurable dose response.

It seems unlikely that a robust QMRA applicable to the broad range of infection scenarios can be usefully derived and applied. The WHO [4] note that ‘it is not possible to consider all water-related human pathogens in a QMRA’. Given the considerable uncertainty at every level of the process the authors believe that the development of a QMRA for *Legionella* spp. is impractical. If this is the case, then it is of little use in establishing the desired goals of informing a water safety risk management plan [4].

Disease caused by other OWI with much more varied routes and sites of infection adds even more complexity. It is worth noting that the majority of water transmitted diseases may be acquired by routes other than ingestion or fecal-oral transmission [51,52,53]. The relatively simple example of *Legionella* spp. as an OWI suggests that QMRA is prohibitively complex. If this is the case, then QMRA for other OWIs with multiple routes of infection is an even less worthwhile proposition. 

## Figures and Tables

**Table 1:** Summary of the uncertainties associated with *Legionella* Quantitative Microbial Risk Assessment (QMRA).

**Problem Formulation** **What are the scope and purpose of the assessment?**	
What hazards?	*Legionella* infections.
Which exposure pathways?	Inhalation and/or aspiration of *Legionella* contaminated water from multiple sources.
Which Health outcomes?	Legionnaire’s disease in susceptible populations, Pontiac fever in general populations.
What certainty is needed for risk management?	This has not been quantified.
**Exposure Assessment** **What is the estimated dose of pathogens for the defined exposure pathway(s)?**	
Pathogen reduction	Achieved by barriers/control measures and managing re-contamination risks–well established control measures for most sources have been developed and applied.
Source concentration	Not defined as there are multiple sources. The relationship between source concentration and exposure has not been quantified due to the diversity of exposure scenarios. A robust and broadly applicable relationship between source concentration and dose has not been quantified.
Magnitude and frequency of exposure	Not quantifiable. Intermittent or occasional use of variable sources by different demographic groups. Many and variable exposure scenarios have been identified.
**Health effects assessment** **What are the expected health effects of the defined hazards?**	
Dose-response	Not quantified in humans. Susceptibility is dependent on the host immune status. Two principle routes of dose delivery exist.
Illness and sequalae	In many cases none. Disease may be self-limited, profound, or fatal. Sequalae range from minor to severe, prolonged and debilitating. Secondary transmission is extremely rare.
Impact on disease burden	DALY * for Years Lived with Disability (YLD) is low (8%). DALY for Years of Life Lost is high (92%).
**Risk characterization** **What are the expected health effects of the estimated dose?**	
Quantification of risk	Reported cases range from 0.5 to 5+: 100,000. Prevalence of disease is much higher in susceptible populations but not quantified. Disease is probably considerably under-reported
Variability and uncertainty analysis	Not calculable for the range of infecting organisms, sources and exposure routes on available evidence.
Sensitivity analysis	Insufficient uncertainty data

* Disability Adjusted Life Years

## References

[1] Buchanan R. (1995). The role of microbiological criteria and risk assessment in haccp. Food Microbiol..

[2] Haas C.N., Rose J.B., Gerba C.P. (1999). Quantitative Microbial Risk Assessment.

[3] Westrell T., Schonning C., Stenstrom T., Ashbolt N. (2004). Qmra and haccp for management of pathogens in wastewater and sewage sludge treatment and reuse. Water Sci. Technol..

[4] World Health Organization (2016). Quantitative Microbial Risk Assessment: Application for Water Safety Management.

[5] Ashbolt N. (2015). Environmental (saprozoic) pathogens of engineered water systems: Understanding their ecology for risk assessment and management. Pathogens.

[6] Petterson S., Ashbolt N. (2016). Qmra and water safety management: Review of application in drinking water systems. J. Water Health.

[7] Whiley H., Keegan A., Giglio S., Bentham R. (2012). Mycobacterium avium complex—The role of potable water in disease transmission. J. Appl. Microbiol..

[8] Armstrong T.W., Haas C.N. (2007). Quantitative microbial risk assessment model for legionnaires’ disease: Assessment of human exposures for selected spa outbreaks. J. Occup. Environ. Hyg..

[9] Armstrong T.W., Haas C.N. (2007). A quantitative microbial risk assessment model for legionnaires’ disease: Animal model selection and dose-response modeling. Risk Anal..

[10] Buse H.Y., Schoen M.E., Ashbolt N.J. (2012). Legionellae in engineered systems and use of quantitative microbial risk assessment to predict exposure. Water Res..

[11] Armstrong T., Haas C. (2008). Legionnaires’ disease: Evaluation of a quantitative microbial risk assessment model. J. Water Health.

[12] Whiley H., Keegan A., Fallowfield H., Ross K. (2014). Uncertainties associated with assessing the public health risk from *Legionella*. Front. Microbiol..

[13] Hamilton K., Haas C. (2016). Critical review of mathematical approaches for quantitative microbial risk assessment (qmra) of *Legionella* in engineered water systems: Research gaps and a new framework. Environ. Sci. Water Res. Technol..

[14] Euser S.M., Pelgrim M., Den Boer J.W. (2010). Legionnaires’ disease and pontiac fever after using a private outdoor whirlpool spa. Scand. J. Infect. Dis..

[15] Ambrose J., Hampton L., Fleming-Dutra K., Marten C., McClusky C., Perry C., Clemmons N., McCormic Z., Peik S., Mancuso J. (2014). Large outbreak of legionnaires’ disease and pontiac fever at a military base. Epidemiol. Infect..

[16] Glick T.H., Gregg M.B., Berman B., Mallison G., Rhodes W.W.J., Kassanoff I. (1978). Pontiac fever. An epidemic of unknown etiology in a health department: I. Clinical and epidemiologic aspects. Am. J. Epidemiol..

[17] Bornstein N., Marmet D., Surgot M., Nowicki M., Arslan A., Esteve J., Fleurette J. (1989). Exposure to legionellaceae at a hot spring spa: A prospective clinical and serological study. Epidemiol. Infect..

[18] Bartram J., Chartier Y., Lee J.V., Pond K., Surman-Lee S. (2007). Legionella and the Prevention of Legionellosis.

[19] Van Heijnsbergen E., Schalk J.A., Euser S.M., Brandsema P.S., den Boer J.W., de Roda Husman A.M. (2015). Confirmed and potential sources of *Legionella* reviewed. Environ. Sci. Technol..

[20] Fisman D.N. (2017). Of Time and the River: How Our Understanding of Legionellosis Has Changed Since 1976.

[21] Rhoads W.J., Garner E., Ji P., Zhu N., Parks J., Schwake D.O., Pruden A., Edwards M.A. (2017). Distribution system operational deficiencies coincide with reported legionnaires’ disease clusters in flint, michigan. Environ. Sci. Technol..

[22] Lesnik R., Brettar I., Höfle M.G. (2016). *Legionella* species diversity and dynamics from surface reservoir to tap water: From cold adaptation to thermophily. ISME J..

[23] Berkelman R.L. (2014). Consideration of prophylactic antibiotic therapy during an outbreak of legionnaires’ disease. Clin. Infect. Dis..

[24] Whiley H. (2016). *Legionella* risk management and control in potable water systems: Argument for the abolishment of routine testing. Int. J. Environ. Res. Public Health.

[25] Crimi P., Macrina G., Grieco A., Tinteri C., Copello L., Rebora D., Galli A., Rizzetto R. (2006). Correlation between *Legionella* contamination in water and surrounding air. Infect. Control Hosp. Epidemiol..

[26] Pourchez J., Leclerc L., Girardot F., Riffard S., Prevot N., Allegra S. (2017). Experimental human-like model to assess the part of viable *Legionella* reaching the thoracic region after nebulization. PLoS ONE.

[27] Berk S.G., Ting R.S., Turner G.W., Ashburn R.J. (1998). Production of respirable vesicles containing live *Legionella pneumophila* cells by two *Acanthamoeba* spp.. Appl. Environ. Microbiol..

[28] Buse H.Y., Ashbolt N.J. (2012). Counting *Legionella* cells within single amoeba host cells. Appl. Environ. Microbiol..

[29] Fields B.S., Benson R.F., Besser R.E. (2002). *Legionella* and legionnaires’ disease: 25 years of investigation. Clin. Microbiol. Rev..

[30] Greig J.E., Carnie J.A., Tallis G.F., Ryan N.J., Tan A.G., Gordon I.R., Zwolak B., Leydon J.A., Guest C.S., Hart W.G. (2004). An outbreak of legionnaires’ disease at the melbourne aquarium, April 2000: Investigation and case-control studies. Med. J. Aust..

[31] Blanchard D.C., Syzdek L.D. (1982). Water-to-air transfer and enrichment of bacteria in drops from bursting bubbles. Appl. Environ. Microbiol..

[32] Strydom D., Küsel R.R., Craig I.K. (2017). When is it appropriate to model transmission of tuberculosis using a dose response model?. IFAC-PapersOnLine.

[33] Edelstein P.H., Metlay J.P. (2009). Legionella pneumophila Goes Clonal—Paris and Lorraine Strain-Specific Risk Factors.

[34] Robertson P., Abdelhady H., Garduño R.A. (2014). The many forms of a pleomorphic bacterial pathogen—The developmental network of *Legionella pneumophila*. Front. Microbiol..

[35] Cirillo J.D., Cirillo S.L., Yan L., Bermudez L.E., Falkow S., Tompkins L.S. (1999). Intracellular growth in *Acanthamoeba castellanii* affects monocyte entry mechanisms and enhances virulence of *Legionella pneumophila*. Infect. Immun..

[36] Cirillo J.D. (1997). Interaction of *Mycobacterium avium* with environmental amoebae enhances virulence. Infect. Immun..

[37] Bouwknegt M., Schijven J.F., Schalk J.A., Husman R., Maria A. (2013). Quantitative risk estimation for a *Legionella pneumophila* infection due to whirlpool use. Risk Anal..

[38] Yamamoto Y., Klein T.W., Newton C.A., Widen R., Friedman H. (1987). Differential growth of *Legionella pneumophila* in guinea pig versus mouse macrophage cultures. Infect. Immun..

[39] Misch E.A. (2016). *Legionella:* Virulence factors and host response. Curr. Opin. Infect. Dis..

[40] Berendt R.F., Young H.W., Allen R.G., Knutsen G.L. (1980). Dose-response of guinea pigs experimentally infected with aerosols of *Legionella pneumophila*. J. Infect. Dis..

[41] Fitzgeorge R., Baskerville A., Broster M., Hambleton P., Dennis P. (1983). Aerosol infection of animals with strains of *Legionella pneumophila* of different virulence: Comparison with intraperitoneal and intranasal routes of infection. Epidemiol. Infect..

[42] Beauté J. (2017). Legionnaires’ disease in europe, 2011 to 2015. Eurosurveillance.

[43] Gregersen P., Grunnet K., Uldum S.A., Andersen B.H., Madsen H. (1999). Pontiac fever at a sewage treatment plant in the food industry. Scand. J. Work Environ. Health.

[44] Van Lier A., McDonald S.A., Bouwknegt M., Kretzschmar M.E., Havelaar A.H., Mangen M.-J.J., Wallinga J., de Melker H.E. (2016). Disease burden of 32 infectious diseases in the netherlands, 2007–2011. PLoS ONE.

[45] Wei S.-H., Chou P., Tseng L.-R., Lin H.-C., Wang J.-H., Sheu J.-N., Liu M.-T., Liu F.-C., Wu H.-H., Lin M.-C. (2014). Nosocomial neonatal legionellosis associated with water in infant formula, taiwan. Emerg. Infect. Dis..

[46] Yiallouros P.K., Papadouri T., Karaoli C., Papamichael E., Zeniou M., Pieridou-Bagatzouni D., Papageorgiou G.T., Pissarides N., Harrison T.G., Hadjidemetriou A. (2013). First outbreak of nosocomial *legionella* infection in term neonates caused by a cold mist ultrasonic humidifier. Clin. Infect. Dis..

[47] Collins S., Afshar B., Walker J., Aird H., Naik F., Parry-Ford F., Phin N., Harrison T., Chalker V., Sorrell S. (2016). Heated birthing pools as a source of legionnaires’ disease. Epidemiol. Infect..

[48] Leitão Filho F.S., Hang Chen H., Ngan D.A., Tam A., Kirby M., Sin D.D. (2016). Current methods to diagnose small airway disease in patients with copd. Expert Rev. Respir. Med..

[49] Den Boer J.W., Yzerman P., Schellekens J., Lettinga K.D., Boshuizen H.C., Van Steenbergen J.E., Bosman A., Van den Hof S., Van Vliet H.A., Peeters M.F. (2002). A large outbreak of legionnaires’ disease at a flower show, the netherlands, 1999. Emerg. Infect. Dis..

[50] Tobin J.O., Dunnill M.S., French M., Morris P.J., Beare J., Fisher-Hoch S., Mitchell R.G., Muers M.F. (1980). Legionnaires’ diseases in a transplant unit: Isolation of the causative agent from shower baths. Lancet.

[51] Gargano J., Adam E., Collier S., Fullerton K., Feinman S., Beach M. (2017). Mortality from selected diseases that can be transmitted by water–united states, 2003–2009. J. Water Health.

[52] Hamilton K.A., Weir M.H., Haas C.N. (2017). Dose response models and a quantitative microbial risk assessment framework for the mycobacterium avium complex that account for recent developments in molecular biology, taxonomy, and epidemiology. Water Res..

[53] Roser D., Van den Akker B., Boase S., Haas C., Ashbolt N., Rice S. (2014). Pseudomonas aeruginosa dose response and bathing water infection. Epidemiol. Infect..

